# Supplementary figures and images for: Immune-associated molecular classification and prognosis signature of sepsis
Source: PLoS One. 2025 Jun 12;20(6):e0326083. doi: 10.1371/journal.pone.0326083 (PMC12161593; doi:10.1371/journal.pone.0326083)

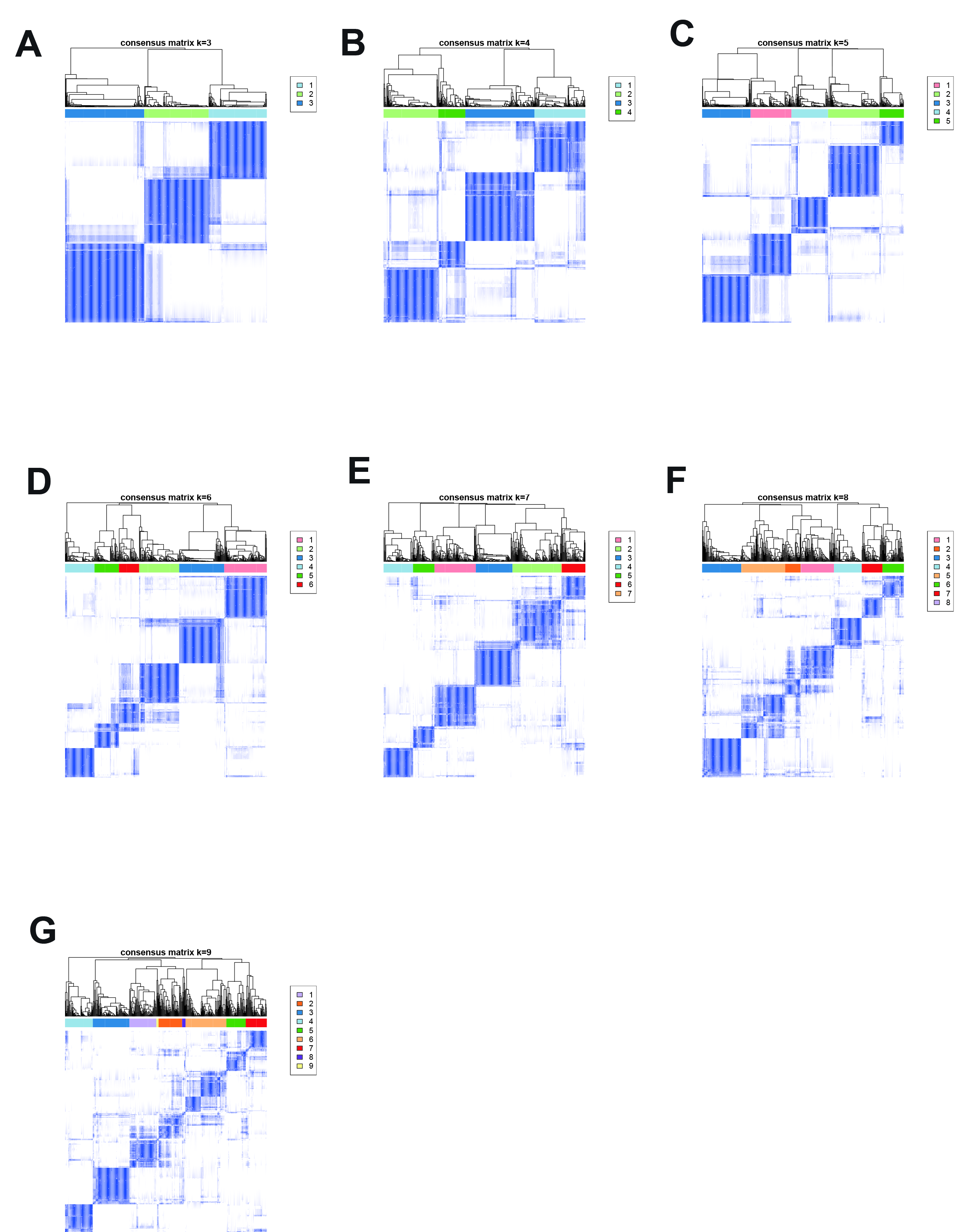

Supplement: S7 Fig — (TIF) [file pone.0326083.s007.tif]
