# Supplementary material for: Immune-associated molecular classification and prognosis signature of sepsis
Source: PLoS One. 2025 Jun 12;20(6):e0326083. doi: 10.1371/journal.pone.0326083 (PMC12161593; doi:10.1371/journal.pone.0326083)
Supplement: Attachment — Submitted filename: Response to Reviewers.docx [file pone.0326083.s012.docx]

**Dear Editor and Reviewers,**

Thank you for your comment. We have revised our manuscript according to these comments. Responses to all the comments are as follows:

**Response to Journal requirements**

When submitting your revision, we need you to address these additional requirements.

**Comment 1.** Please ensure that your manuscript meets PLOS ONE's style requirements, including those for file naming. The PLOS ONE style templates can be found athttps://journals.plos.org/plosone/s/file?id=wjVg/PLOSOne_formatting_sample_main_body.pdfandhttps://journals.plos.org/plosone/s/file?id=ba62/PLOSOne_formatting_sample_title_authors_affiliations.pdf

**Response 1:** Yes, the manuscript has been prepared according to the style requirements.

**Comment 2.** Please note that PLOS ONE has specific guidelines on code sharing for submissions in which author-generated code underpins the findings in the manuscript. In these cases, we expect all author-generated code to be made available without restrictions upon publication of the work. Please review our guidelines at https://journals.plos.org/plosone/s/materials-and-software-sharing#loc-sharing-code and ensure that your code is shared in a way that follows best practice and facilitates reproducibility and reuse.

**Response 2:** Yes, we have uploaded all codes as supplementary materials.

**Comment 3.** For studies involving third-party data, we encourage authors to share any data specific to their analyses that they can legally distribute. PLOS recognizes, however, that authors may be using third-party data they do not have the rights to share. When third-party data cannot be publicly shared, authors must provide all information necessary for interested researchers to apply to gain access to the data. (<https://journals.plos.org/plosone/s/data-availability#loc-acceptable-data-access-restrictions>) For any third-party data that the authors cannot legally distribute, they should include the following information in their Data Availability Statement upon submission:1) A description of the data set and the third-party source. 2) If applicable, verification of permission to use the data set. 3) Confirmation of whether the authors received any special privileges in accessing the data that other researchers would not have. 4) All necessary contact information others would need to apply to gain access to the data

**Response 3:** Yes, we have updated these descriptions in the Data Availability Statement as follows: The expression levels of immune-related genes from sepsis patients can be available from the GEO without any special privileges (GSE65682: https://www.ncbi.nlm.nih.gov/geo/query/acc.cgi?acc=GSE65682), which is publicly for everyone. The validation dataset and original codes are within the manuscript and its Supporting Information files.

**Comment 4:** PLOS requires an ORCID iD for the corresponding author in Editorial Manager on papers submitted after December 6th, 2016. Please ensure that you have an ORCID iD and that it is validated in Editorial Manager. To do this, go to ‘Update my Information’ (in the upper left-hand corner of the main menu), and click on the Fetch/Validate link next to the ORCID field. This will take you to the ORCID site and allow you to create a new iD or authenticate a pre-existing iD in Editorial Manager.

**Response 4:** Yes, we have added the ORICID for corresponding author.

**Comment 5.** Please review your reference list to ensure that it is complete and correct. If you have cited papers that have been retracted, please include the rationale for doing so in the manuscript text or remove these references and replace them with relevant current references. Any changes to the reference list should be mentioned in the rebuttal letter that accompanies your revised manuscript. If you need to cite a retracted article, indicate the article’s retracted status in the References list and include a citation and full reference for the retraction notice.

**Response 5:** Yes, we have reviewed all reference lists and made sure they are right. The added references have been mentioned.

**Response to Reviewer #1**

This is a very complex study involving a considerable amount of work with clinically important objectives. The manuscript is well written, and the discussion is argued convincingly. The discussion of the pros and the limitations of the study were insightful. **Comment 1:** Could the authors consider adding further specific details about the findings of other similar prior studies in the discussion?

**Response 1:** Thank you for your comment. We have the following discussion and make some comparisons with a previous study in data usage and analysis methods, added analysis content, and the advantages and disadvantages of the model: A recent study also analyzed the associations between immune-related genes and clinical prognosis or the immune microenvironment in sepsis [17]. There are evident differences between our research and previous studies that we need to address. First, although we utilized the same dataset, our research diverges from previous studies in terms of data processing strategies. While prior studies divided the GEO dataset into training and validation sets for model construction, our study used the GEO dataset exclusively as a training set and incorporated an independently collected validation cohort. Secondly, our research performed an unsupervised clustering analysis on the GEO dataset, identifying two molecular subtypes with significantly distinct clinical prognoses, biological functions, and immunologic backgrounds, which had not been done in earlier studies. Furthermore, our model differs from those developed in prior research; whereas previous studies created a prognostic model comprising seven immune-related genes with a consistency index of 0.78, our model includes twenty-one immune-related genes and achieves a consistency index of 0.83. In terms of predictive ability, our model’s predictive ability was relatively high. Finally, our research established a personalized risk scoring system based on risk scores and estimated the mortality risk for patients at each critical time point. Additionally, we connected the molecular subtypes of patients with sepsis to the prognostic risk patterns, forming a “molecular subtype-risk identification-risk assessment” system. Our study not only enhances but also expands upon prior research, providing robust supporting data for validation.

We then added some other gene prognosis model studies: Some other gene prognosis models were also developed. Yang et al developed a nine-gene model and the AUCs were 0.83, 0.81, and 0.77 at 7, 14 and 21 days. But the internal and external validation datasets were lacking for this study.

17.	Chen ZH, Zhang WY, Ye H, Guo YQ, Zhang K, Fang XM. A signature of immune-related genes correlating with clinical prognosis and immune microenvironment in sepsis. Bmc Bioinformatics. 2023;24(1):20. http://doi.org/10.1186/s12859-023-05134-1

31. Huang C, Liu Z, Guo Y, Wang W, Yuan Z, Guan Y, et al. scCancerExplorer: a comprehensive database for interactively exploring single-cell multi-omics data of human pan-cancer. Nucleic Acids Res. 2025;53(D1): D1526-35. http://doi.org/10.1093/nar/gkae1100

32.	Peng Y, Wu Q, Ding X, Wang L, Gong H, Feng C, et al. A hypoxia- and lactate metabolism-related gene signature to predict prognosis of sepsis: discovery and validation in independent cohorts. Eur J Med Res. 2023;28(1):320. http://doi.org/10.1186/s40001-023-01307-z

33.	Liang S, Xing M, Chen X, Peng J, Song Z, Zou W. Predicting the prognosis in patients with sepsis by a pyroptosis-related gene signature. Front Immunol. 2022; 13:1110602. http://doi.org/10.3389/fimmu.2022.1110602

**(1) I have some minor questions**

**Comment 2:** Could the authors expand in the discussion regarding any paradoxes that there may be in the biological function and immune status (immune cells and checkpoint genes) seen between cluster A (Figures 5B, C&D) and the high-risk groups (Fig 9C, D & E).

**Response 2:** Thank you for your comment. We have added the following discussion: In sepsis, both the function and the number of various immune cells undergo significant changes, leading to an immunosuppressive state. These alterations include shifts in cell counts, functional impairments, and imbalances in the secretion of pro-inflammatory and anti-inflammatory factors. Together, these disruptions contribute to immune system dysfunction, which adversely impacts the clinical outcomes of patients. This study found that patients in cluster A had a worse prognosis compared to those in cluster B. Immune infiltration analysis revealed that most immune cells and their functions were suppressed in cluster A patients, and the expression of immune checkpoint-related genes was relatively low, supporting the notion of immune system suppression in sepsis. Similar trends were observed in high-risk and low-risk groups. However, some immune cells and functions, such as iDCs, macrophages, and Tregs, were elevated in patients with a poor prognosis, particularly in cluster A. This may be linked to their specific roles: iDCs, although they have a strong capacity for antigen uptake, are functionally inactive and have a limited ability to activate T-cell responses; macrophages primarily regulate non-specific immunity and help maintain immune tolerance. Their increased presence may represent a stress response. Additionally, we observed increased expression of two immune checkpoint genes, CD200R1 and LAIR1, both of which are immunosuppressive. This upregulation is associated with the immunosuppressive effects seen in sepsis patients. In the high-risk group, LAIR1 was also elevated, as were immunosuppressive genes CD276 and PDCD1LG2, which is consistent with immunosuppressive status in sepsis patients.

**Comment 3:** Could the authors expand in the discussion their hypotheses regarding the poor prognostic performance of variables that have been associated with poor prognosis in prior phenotypic studies e.g., age, sex. Also, it may be interesting to expand on the poor performance of "endotype" in both the training and validation datasets.

**Response 3:** Thank you for your comment. We have added the following discussion: In multivariate analyses, we found confirmed that the risk score based on immune-related genes was an independent prognosis factor in both training and validation datasets. We also found that age was independently associated with poor prognosis in sepsis. Older adults are immunocompromised, making them more vulnerable to sepsis, and their prognosis tends to be poorer. We did not find the correlation of gender with prognosis in sepsis patients. Previous study suggested that gender may influence age-associated sepsis outcomes. We assume that there mab be interactions between age and gender, which need to be further confirmed [37]. We also found no association between endotype and prognosis in sepsis patients. Endotype is genotyped based on the genome, but sepsis is characterized by rapid disease progression, requiring quick clinical decision-making. Genotype-based endotyping may therefore have certain time lags. Additionally, the variation in patient characteristics could play a role. For instance, sepsis patients often present with immunosuppression, rapid disease progression, and the administration of specific treatments, all of which may influence the relationship between molecular typing and prognosis.

**I have some minor suggestions re: visualization of the data:**

**Comment 4:** Figure 2A - the labelling is not clearly visualized for a number of the genes in this figure, could the resolution of this figure be optimized? Could this be achieved by reducing the number of genes in the figure to only those with a cox p value of 0.05 or less? Could changing the colour of the negative correlations from pink also improve the resolution of correlations.

**Response 4:** Thank you for your comment. We have revised Figure 2A.

**Comment 5:** Maintaining consistency of the colors representing clusters A and B in all figures and graphs could be considered to help the reader easily identify the relevant cluster.

**Response 5:** Yes, we have checked all Figures and maintained the consistency of colors for cluster A and B. We marked the red for cluster A and blue for cluster B.

**Comment 6:** Is the unit for K-M (Fig 2F) intended to be days rather than years?

**Response 6:** Yes, the unit of K-M in Figure 2F should be days. We have revised this.

**Response to Reviewer #2**

Overall, an informative and interesting study. The study provides valuable insights into sepsis prognosis using immune-related molecular subtypes. The use of machine learning (LASSO regression, Cox regression) enhances predictive modelling for clinical applications.

**Comment 1:** Introduction: Agree regarding the importance of monocytes and neutrophils as effectors of innate responses during sepsis – required for microbial clearance and recruiting other immune cells. CD4 T cells are important for modulation but I recommend mentioning the role of regulating immune responses through cells such as Tregs that are needed to suppress exaggerated cytokine responses. CD8 effectors will be important here too. Also, dysfunction in these cells may lead to, for example, inappropriate interferon responses that help locally (paracrine signaling) as seen in severe COVID-19 infection. I recommend that if the author mentions or is referencing a specific study (e.g., line 98) then please summarize the findings for the benefit of the reader before the critique.

**Response 1:** Thank you for your comment. We have added the following descriptions: Previous studies have focused on the effect of individual genes on sepsis. Arunachalam et al. investigated the role of P2Y2 purinergic receptors in liver injury and sepsis. They found that mice lacking P2Y2 receptors exhibited reduced inflammation, less liver damage, and improved survival in response to both inflammatory liver injury and sepsis. These P2Y2 knockout mice also maintained normal serum arginine levels, preventing immune dysregulation and increased bacteremia seen in wild-type mice. The findings suggest that P2Y2 receptors play a crucial role in the pathophysiology of liver injury and sepsis, and targeting this receptor could potentially mitigate cytokine storms and improve outcomes in sepsis [14]. Liu et al. found that FCGR2C was the only gene differentially expressed between survivors and non-survivors in a cohort of 81 septic patients. FCGR2C was more predictive than the SOFA score in evaluating the prognosis of septic patients [15]. Jian et al. found that the level of UCP2 in blood cells of sepsis patients was significantly higher than in healthy controls, both at the mRNA and protein levels. UCP2 in blood cells may serve as a specific biomarker for sepsis, and its level is positively correlated with the severity of sepsis. [16]. These studies on individual genes have been insufficient in fully elucidating the complex pathophysiological processes of sepsis.

14.	Arunachalam AR, Samuel SS, Mani A, Maynard JP, Stayer KM, Dybbro E, et al. P2Y2 purinergic receptor gene deletion protects mice from bacterial endotoxin and sepsis-associated liver injury and mortality. Am J Physiol-Gastr L. 2023;325(5):G471-91. http://doi.org/10.1152/ajpgi.00090.2023

15.	Liu S, Zhang YL, Zhang LY, Zhao GJ, Lu ZQ. FCGR2C: An emerging immune gene for predicting sepsis outcome. Front Immunol. 2022;13:1028785. http://doi.org/10.3389/fimmu.2022.1028785

16.	Jiang ZM, Yang QH, Zhu CQ. UCP2 in early diagnosis and prognosis of sepsis. Eur Rev Med Pharmaco. 2017;21(3):549-53.

**Comment on Methods and results**

**Comment 2:** The paper utilizes a well-structured bioinformatics approach (WGCNA, GSVA, and immune infiltration analysis). Inclusion of an independent validation cohort strengthens the reliability of findings. The training dataset was a combination of GSE65682 (where there was complete data) and new recruits. It would be good to include how sepsis was diagnosed (criteria) and these participants. Also, a demographic table to understand the cohort – as a training dataset the readers will want to know how relevant they were, was there a risk of bias? Were there differences in demographics or clinical characteristics between the training and validation sets? What was the mortality rates (given the eventual risk groups are based on progression to death or risk based on another prognosis tool?). A table summarizing this would add value.

**Response 2:** Yes, we have added the diagnosed criteria for sepsis. According to the Sepsis-3 criteria: The diagnosis of sepsis is based on the SOFA (Sequential Organ Failure Assessment) score, which assesses the extent of organ dysfunction. A ≥2 increase in SOFA score indicates sepsis[18]. A demographics table between training and validation sets was also added (Table 1). The mortality rates were also reported.

18. Seymour CW, Liu VX, Iwashyna TJ, Brunkhorst FM, Rea TD, Scherag A, et al. Assessment of Clinical Criteria for Sepsis: For the Third International Consensus Definitions for Sepsis and Septic Shock (Sepsis-3). Jama-J Am Med Assoc. 2016;315(8):762-74. http://doi.org/10.1001/jama.2016.0288

**Comment 3:** It would help the reader if there were a list of genes included used to associate with the different cell types and subsets – perhaps in a table.

**Response 3:** Yes, the genes lists were provided. All immune-related genes lists (S3 Table), differentially expressed genes between cluster A and cluster B (S6 Table), and differentially expressed genes between high-risk and low-risk groups (S9 Table) have been provided.

**Comment 4:** The k-value selection for clustering is mentioned, but further justification would strengthen the argument. Why was k=2 chosen? Was this the optimum value (the text suggests this was not assessed)? How was cluster stability assessed?

**Response 4:** Thank you for your comment. The K value selection is based on the following descriptions: In Methods: Non-negative matrix factorization clustering was performed, and the cophenetic correlation coefficients and cumulative distribution function were calculated to determine the optimal k value. The consensus matrix legend was used to determine the optimal k-value, while empirical cumulative distribution function plots were used to display the consensus distributions for each k value. The K value will be selected when the cophenetic correlation coefficient is higher with flatter cumulative distribution function and significant differences in prognosis. We also provide the matrix heatmaps for other k values (k>2) in S5 Fig.

In results: The cophenetic correlation coefficient is the maximum, and cumulative distribution function is flatter when k=2. Importantly, the subtypes show significant differences in prognosis. When k>2, the consensus matrix heatmap shows unsharp and blur boundaries. A k value of 2 was eventually chosen as the optimal number.

**Comment 5:** In WGCNA, the soft thresholding power selection (R² maximisation) is briefly mentioned but should be explained further.

**Response 5:** Yes, we added the following descriptions in the methods: When constructing a gene co-expression network, in order to make the network resemble a scale-free network, an appropriate β value, also known as the soft threshold parameter, must be selected. This value determines how the connection weights in the network increase as the correlation between genes strengthens. Generally, the goal in selecting this value is to make the relationship between the network's average connectivity (k) and the logarithm of the number of genes (log(k)) appear linear on a log-log plot. It is also desirable for the degree distribution of the network to exhibit scale-free distribution characteristics. To select the optimal soft threshold, the scale-free topology fit index under different β values can be observed. When this index approaches or reaches 0.9, it indicates that the β value is appropriate.

**Comment 6:** The study uses HR (hazard ratio) and confidence intervals (CI) for survival analysis. However, was a power calculation performed to ensure sample size sufficiency? Were multiple testing corrections (e.g., Bonferroni, FDR) applied for differential expression and enrichment analyses?

**Response 6:** Thank you for your comment. The power is 92.6 when α=0.05, N=479, Log HR=0.1 (risk score) using PASS software. The adjusted P values were used for differential expression and enrichment analyses. We have added these descriptions in methods and results.

**Comment 7:** In general, the bioinformatics workflow is well structured. Including: unsupervised clustering (ConsensusClusterPlus) to identify molecular subtypes, WGCNA (Weighted Gene Co-expression Network Analysis) used for gene module detection, Gene Ontology (GO) and KEGG pathway analyses for biological function, and immune infiltration analysis (CIBERSORT) for immune cell distributions.

**Response 7:** Thank you for your comment.

**Comment 8:** The authors have stratified further by assessing the tool used for independent variables that will influence mortality such as age, but what about the sepsis causing organism since this may affect pathogenesis and progression to severe sepsis?

Response 8: Thank you for your valuable comment. Unfortunately, the database we used for this study did not collect information regarding the specific organisms responsible for sepsis. As a result, we were unable to assess the impact of different sepsis-causing pathogens on the pathogenesis and progression to severe sepsis. We acknowledge that this is an important factor that could influence outcomes, and we recommend that future studies with more comprehensive datasets consider incorporating pathogen-specific data to better understand these dynamics. But we added some study limitations in the discussion.

**Comment 9:** Discussion: This is comprehensive, and the authors have appreciated the study limitations and future directions.

**Response 9:** Thank you for your comment.
