# Supplementary material for: Immune-associated molecular classification and prognosis signature of sepsis
Source: PLoS One. 2025 Jun 12;20(6):e0326083. doi: 10.1371/journal.pone.0326083 (PMC12161593; doi:10.1371/journal.pone.0326083)
Supplement: Attachment — Submitted filename: Response_to_Reviewers_auresp_2.docx [file pone.0326083.s013.docx]

**Response to Editorial**

**Comment:** Journal Requirements: Please review your reference list to ensure that it is complete and correct. If you have cited papers that have been retracted, please include the rationale for doing so in the manuscript text or remove these references and replace them with relevant current references. Any changes to the reference list should be mentioned in the rebuttal letter that accompanies your revised manuscript. If you need to cite a retracted article, indicate the article’s retracted status in the References list and also include a citation and full reference for the retraction notice.

**Response:** Yes, we have checked all references one by one, and there are no retracted papers in the references lists.

**Response to Reviewers**

Reviewer #1: This is a very interesting and impactful study which merits publication. It has important objectives, and the findings provide the basis for further investigation of sepsis molecular signatures, immune-related pathways and individualized prognosis for sepsis. The points raised following the last review have been responded to thoughtfully. Feedback in overview: The impact of this study may be communicated more robustly following some minor revisions. The manuscript is written in standard English and overall, is very well written, particularly the abstract and the last part of the discussion. However, in some areas it has less clarity and is less fluent, not uncommon when a manuscript has been formulated by multiple co-authors. Given that manuscripts are not copyedited by PLOS ONE, I would recommend that the authors consider further self-edits to address this. Also, I recommend the addition of further relevant citations to support statements regarding prior knowledge e.g., in the introduction and discussion.

Introduction:

**Comment 1:** Line 70 - As this reference is of a study looking at neonatal infections, additional relevant references are recommended to support this statement.

**Response 1:** Yes, we have updated this reference citation using the following one:

3. Rudd KE, Johnson SC, Agesa KM, Shackelford KA, Tsoi D, Kievlan DR, et al. Global, regional, and national sepsis incidence and mortality, 1990-2017: analysis for the Global Burden of Disease Study. Lancet. 2020;395(10219):200-11. http://doi.org/10.1016/S0140-6736(19)32989-7

**Comment 2:** Line 73 - An additional reference relating to findings in humans would be recommended here.

**Response 2:** Yes, we have added the following reference:

6. Apitzsch S, Larsson L, Larsson AK, Linder A. The physical and mental impact of surviving sepsis - a qualitative study of experiences and perceptions among a Swedish sample. Arch Public Health. 2021;79(1):66. http://doi.org/10.1186/s13690-021-00585-

**Comment 3:** Line 107 - Regarding FCGR2C - I presume upregulation of this gene was seen in non-survivors but I would recommend clarification in the statement.

**Response 3:** Yes, we revised these descriptions as follows: Liu et al. found that FCGR2C was the only down-regulated gene differentially expressed between survivors and non-survivors in a cohort of 81 septic patients.

**Comment 4:** Line 112 - It would be helpful for the authors to expand re: what the study aims of the cited studies were at this point. This would help clarify the comparisons that the authors make with their study design. I note that they return to this later on in the manuscript but feel that this would improve the fluency of the manuscript if the topic was also addressed prior to leading into the advantages of the authors' study design.

**Response 4:** Thank you for your advice. What we would like to clarify here is that previous studies focused on individual genes were unable to fully explain the complex physiological and pathological processes of sepsis. Although there have been studies on immune gene sets and sepsis prognosis, our research differs from previous studies in several important aspects such as molecular subtypes, immune-cell infiltration, individual risk evaluation, and independent validation cohort data, which is a general description. Because it is impossible for us to directly present our specific results in the Introduction Section. Later in the Discussion Section of the article, we present the specific details of these differences.

**Comment 5:** Line 152& 163/ Table 1 - This table is a valuable inclusion in the manuscript and the authors appropriately highlight the presence of missing data. I would recommend that they state clearly how they managed the missing data. What statistical analyses were performed to account for the missing data? Also, this Table might be better placed in the "Results" section. Additionally, there is a high percentage of missing data in the training dataset e.g., unknown classification for thrombocytopenia in the training group etc., Can the authors discuss re: their confidence in the significance of the statistical differences seen in the parameters (between the training and validation sets) when a large portion of the categorization is unknown. I would also recommend expanding on this point in the discussion re: study limitations e.g., on how this could affect the results interpretation of the data (particularly the clinical prognostic tools).

**Response 5:** Thank you for your advice. Table 1 has been placed in the Results Section.

For missing data, we handle the missing data as the third type of variable to make full use of this data. We further performed the stratified analyses for these variables. We still found significant differences in survival outcomes between high-risk and low-risk group (revised Figure 7). We also added the following descriptions in the study limitations: Second, the variables (pneumonia, thrombocytopenia, infection type, and diabetes) had some missing data. We included these variables in the multivariate cox regression to make full use of this dataset. This may generate some information bias. The stratified analyses in the missing data and results from the validation dataset weaken the effects of missing data on results.

**Comment 6:** Line 282 - Figure 1. I would recommend checking the table for typos (Kaplan-Meier) and consistency of content. The sample number appears differently in the table and in the text (890 - line 282; 802 - line 141).

**Response 6:** Yes, we have revised this. It should be 802.

**Comment 7:** Line 375 - Figures - 7A-7N - The unit on the K-M curves is years and is likely to be a typo. Line 385 - Figure 7 - I would recommend changing the labelling on some of these graphs to aid the reader. The legend is well written. However, on the graphs, it would be recommended to change 7 E/F; I/J; M/N from yes/ no to the parameter being evaluated e.g., ICU-acquired infection or Non-ICU-acquired infection etc.

**Response 7:** Yes, we have revised the unit in Figure 7. It should be Days. The labels were also revised according to the figure legends.

**Comment 8:** Discussion: I would recommend including citations to support statements made. I would recommend clarification of the sentences in Lines 448 & 512. They appear to potentially contradict each other.

**Response 8:** Yes, we revised this description as follows: Immune infiltration analysis showed that Cluster B had higher immune cell counts (CD8+ T cells, NK cells) than Cluster A, and immune function levels were also higher in Cluster B than in Cluster A.

**Comment 9:** I would also recommend clarification of the statement in Line 512 regarding immune checkpoint-related genes being relatively low. Often an increase in expression of checkpoint inhibitory markers (at the protein level) is associated with immune suppression. A suitable citation would be recommended here. Also, does this contradict statements made later e.g., Line 607-608?

**Response 9:** Yes, for line 512, we revised this description as follows: Immune infiltration analysis revealed that most immune cells and their functions were suppressed in cluster A patients, and the expression of immune checkpoint inhibitory genes was relatively elevated, supporting the notion of immune system suppression in sepsis. The following reference was cited.

36. Liu YC, Shou ST, Chai YF. Immune checkpoints in sepsis: New hopes and challenges. Int Rev Immunol. 2022;41(2):207-16. http://doi.org/10.1080/08830185.2021.1884247

**Comment 10:** I would also recommend that the authors include further relevant citations throughout the discussion to support statements e.g., line 512 E.g., Line 517-519 - I would recommend adding supportive citations.

**Response 10: Yes, the following references were cited:**

37.Guermonprez P, Valladeau J, Zitvogel L, Thery C, Amigorena S. Antigen presentation and T cell stimulation by dendritic cells. Annu Rev Immunol. 2002;20:621-67. <http://doi.org/10.1146/annurev.immunol.20.100301.064828>

38. Luo B, Gan W, Liu Z, Shen Z, Wang J, Shi R, et al. Erythropoeitin Signaling in Macrophages Promotes Dying Cell Clearance and Immune Tolerance. Immunity. 2016;44(2):287-302. http://doi.org/10.1016/j.immuni.2016.01.002

**Comment 11:** Line 527 - This section is very interesting. The impact of this study would be enhanced by the authors contrasting the interpretation of the findings of the studies mentioned (with their study) and the potential implications.

**Response 11:** Thank you for your comment. We added the following descriptions as follows: In our study, we also obtained two subtypes (cluster A and cluster B) based on immune-related genes, and the cluster A had poorer prognosis than cluster B. Our study provided a different perspective for sepsis subtypes.

**Comment 12:** Line 580 - I would recommend supporting this statement by adding the specific details from the study findings.

**Response 12:** Yes, these statements have been revised as follows: we observed that the levels of Th1 and Th2 cells were decreased in high-risk group.

**Comment 13:** Line 584 - I would recommend clarification of the differences seen where it is stated that the expression is "abnormal".

**Response 13:** Yes, we have revised these descriptions using “increased” or “decreased” instead of “abnormal”

**Comment 14:** Line 594-596 - This section would benefit from additional context here.

**Response 14:** A previous study investigated the leukocyte functions before and after passive blocking of PD-1 and PD-L1 with antibodies. White blood cells were derived from patients with sepsis, critically ill patients without sepsis and healthy controls. They

found that neutrophil and monocyte functions in patients with sepsis are decreased, which is related to CD8+ T cell- and NK cell-specific PD-1 and PD-L1 expression [50]. Interestingly, decreased CD8+ T cell effector function (such as IFNγ production capacity) was associated with elevated PD-L1 expression in neutrophils. After the application of anti-PD-1 or PD-L1 antibodies, the function of neutrophils and monocytes was restored, leukocyte apoptosis was reduced in patients with sepsis, and the function of T cell effects was also significantly restored, manifested by a significant increase in IFN-γ and IL-2 production [51]. This provides evidence for T cell exhaustion in patients with sepsis, but it is reversible.

**Comment 15:** Line 600 - This is very good to include but may benefit from being included earlier in the discussion, before the earlier mention of immune checkpoint molecules.

**Response 15:** Yes, we moved these descriptions in earlier section in the discussion.
